# Primitive ovarian carcinosarcoma: a clinical and radiological analysis of five cases

**DOI:** 10.1186/s13048-020-00728-2

**Published:** 2020-10-28

**Authors:** Qiong Xu, Xiaofei Zhang, Yu Zou

**Affiliations:** 1 Department of Radiology, Women’s Hospital School of Medicine Zhejiang University, No. 1 Xueshi Road, Hangzhou, 310006 Zhejiang Province China; 2 Department of Pathology, Women’s Hospital School of Medicine Zhejiang University, No. 1 Xueshi Road, Hangzhou, 310006 Zhejiang Province China

**Keywords:** Ovarian carcinosarcomas (OCS), Computed tomography (CT), Magnetic resonance imaging (MRI), Surgery, Chemoradiotherapy

## Abstract

**Background:**

Ovarian carcinosarcomas (OCS) are very rare tumors composed of a mixture of carcinomatous and sarcomatous elements. There have been only scattered case studies that have described the imaging findings. In order to improve the awareness of this rare tumor, this study aimed to analyze the clinical and imaging features of five cases of OCS confirmed by surgical pathologic evaluation.

**Methods:**

This retrospective study includes five OCS patients diagnosed and treated at our institute. The clinical course and imaging findings of all patients were retrospectively analyzed. The patients were 31 to 59 years of age. All five patients underwent CT scans, two underwent MRI scans.

**Results:**

The five patients have no specific symptoms. Four patients had elevated CA 125 levels and three patients had elevated CA 153 levels. All patients had unilateral tumors, four in the left ovary, one in the right ovary. The largest transverse diameter of the tumors ranged from 11 cm to 14 cm. Two tumors showed solid masses with unequally sized cystic areas or necrosis, one showed a multilocular cystic mass with a large solid protrusion, two tumors showed a larger cystic mass with multiple mural nodules. The solid components of the tumors demonstrated restricted diffusion (the average ADC value being 998 mm^2^/s and 1102 mm^2^/s, respectively), and showed moderate or obvious enhancement. All five patients were treated by surgical resection and adjuvant chemotherapy. One patient is currently undergoing post-operative chemotherapy 1 month after operation and clinical stable. Three patients survived and showed no obvious recurrence and / or metastasis in follow-up from 9 to 59 months. One patient died from recurrence and metastasis.

**Conclusions:**

OCS are rare and demonstrate variable CT and MRI morphological appearances. Due to the heterogeneous nature and very low morbidity of OCS, combination of careful analysis of imaging findings and clinical features might be useful for a more accurate diagnosis of OCS.

## Background

Ovarian carcinosarcomas (OCS), also called malignant mixed müllerian tumor (MMMT), are rare and aggressive tumors, accounting for less than 1% of all ovarian malignancies. Moreover, OCS have been reported to have poor prognosis. Computed tomography (CT) and magnetic resonance imaging (MRI) are essential to assess the size, extent of the tumor, evidence of metastasis, and infiltration to the surrounding tissues. Moreover, CT and / or MRI can provide more comprehensive information in the diagnosis and therapy. To date, numerous studies have focused on histopathological characteristics, clinical manifestations and prognosis of OCS [1, 2], by contrast, there have been only scattered case studies that have described the imaging findings [3–7]. The purpose of the present study was to analyze the clinical features, CT and MRI characteristics of OCS, and to describe the pathological features and prognosis.

## Results

### Clinical findings

The clinical features of the five patients were reviewed and the findings summarized in Table 1. The age of the patients was from 31 to 59 years with the mean age of 45.4 years. None of the patients had received preoperative therapy. The five patients have no specific symptoms, three presented with abdominal pain, one showed weakness and weight loss, one was asymptomatic and the mass was incidentally discovered.

**Table 1:** Clinical features

No./Age	Symptom	Laboratory data	Treatment	FIGO	Follow up		
					FD (month)	Recurrence	Result
1/43	Asymptomatic	CA125:764.8 U/ml; CA153:85.6 U/ml;	WE+CT	IIB	52	N	CR
2/31	Abdominal pain	CA125:94.4 U/ml; CA153:29.5 U/ml;	WE+CT	IC2	9	N	CR
3/50	Weakness and weight loss	CA125:2158 U/ml; CA199:149.7 U/ml;	WE+CT	IV	59	N	CR
4/44	Abdominal pain	Normal	WE+CT	IIB	6	Y	PD (death)
5/59	Abdominal pain	CA125:225.8 U/ml; CA153:64.2 U/ml;	WE+CT	IIIC	1	N	CR

*CA125* Cancer antigen 125, *CA153* Cancer antigen 153, *CA199* Cancer antigen 199, *WE* Wide excision, *CT* Chemotherapy, *FD* Follow-up, *N* No, *Y* Yes, *CR* Complete response, *PD* Progressive disease

### Imaging findings

**Fig. 1:**
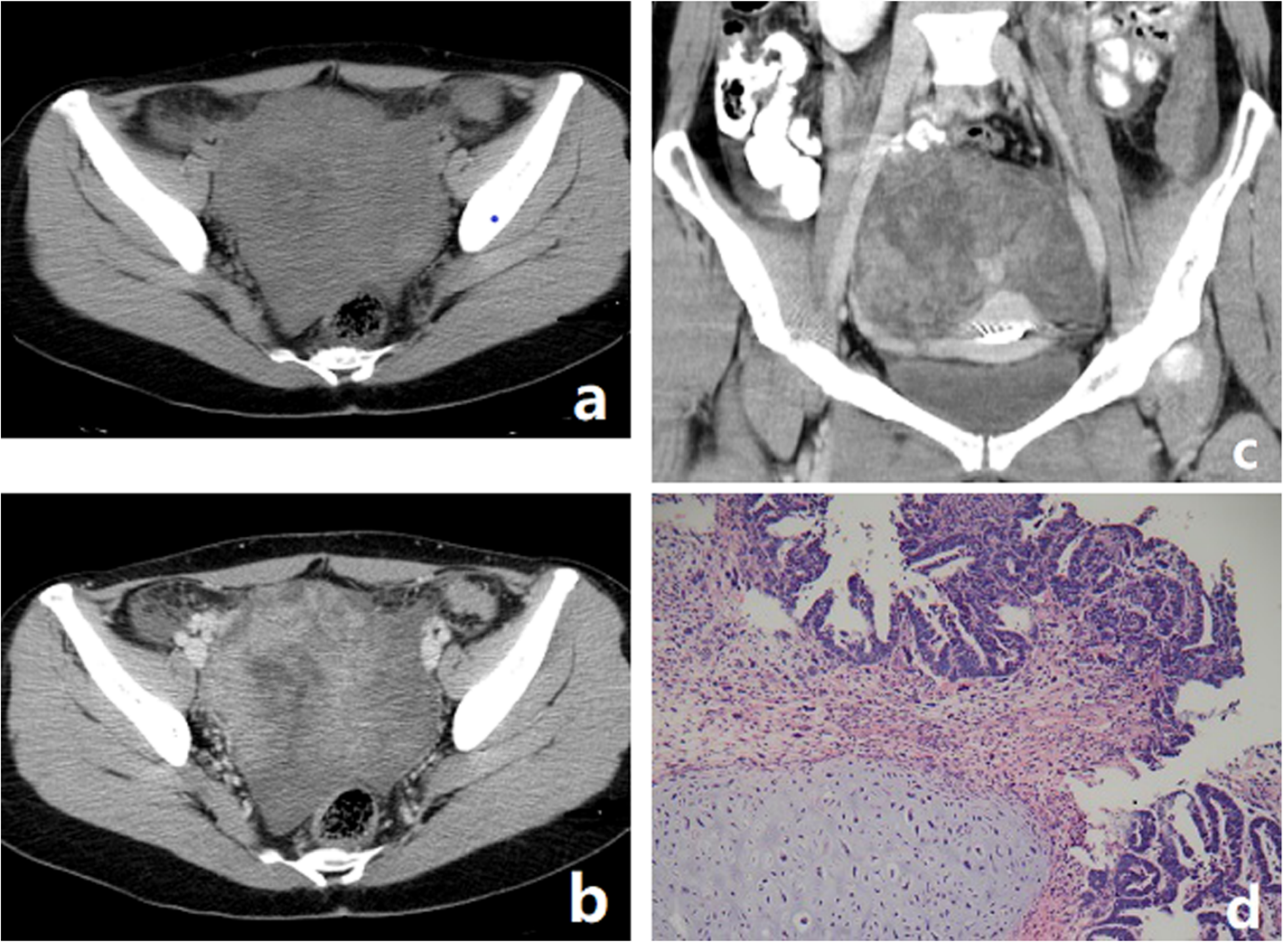
OCS in a 50-year-old woman with weakness and weight loss for 1 month. **a** Axial CT showed a predominantly solid mass with unequally sized cystic areas or necrosis. **b**-**c** After the administration of contrast agent, the tumor parenchyma can appear heterogeneous moderate enhancement. **d** Photomicrograph showing the tumor containing both carcinomatous and sarcomatous elements. (HE 20&10). The heterologous mesenchymal elements contain cartilage

**Fig. 2:**
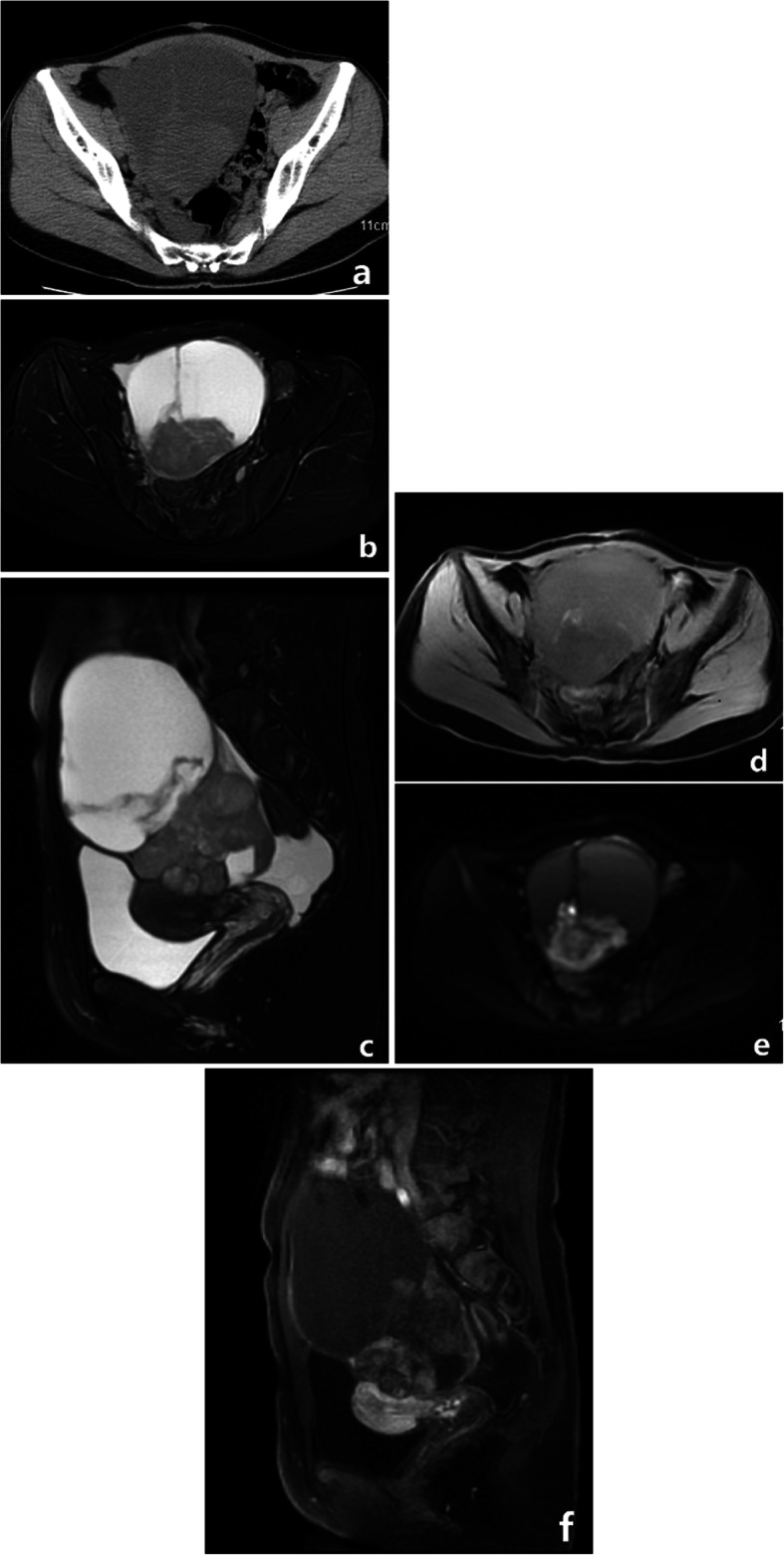
OCS in a 43-year-old woman with abdominal pain. **a** Axial CT showed a multilocular cystic mass with a large solid protrusion. The solid component was iso-dense on CT and slightly high SI on fat saturation T2WI (**b**, **c**), slightly low SI on fat saturation T1WI (**d**), and high SI on DWI (**e**), along with hemorrhagic patchy high SI on fat saturation T1WI insider the tumor. **f** After the administration of contrast agent, the tumor parenchyma can appear heterogeneous moderate enhancement

**Fig. 3:**
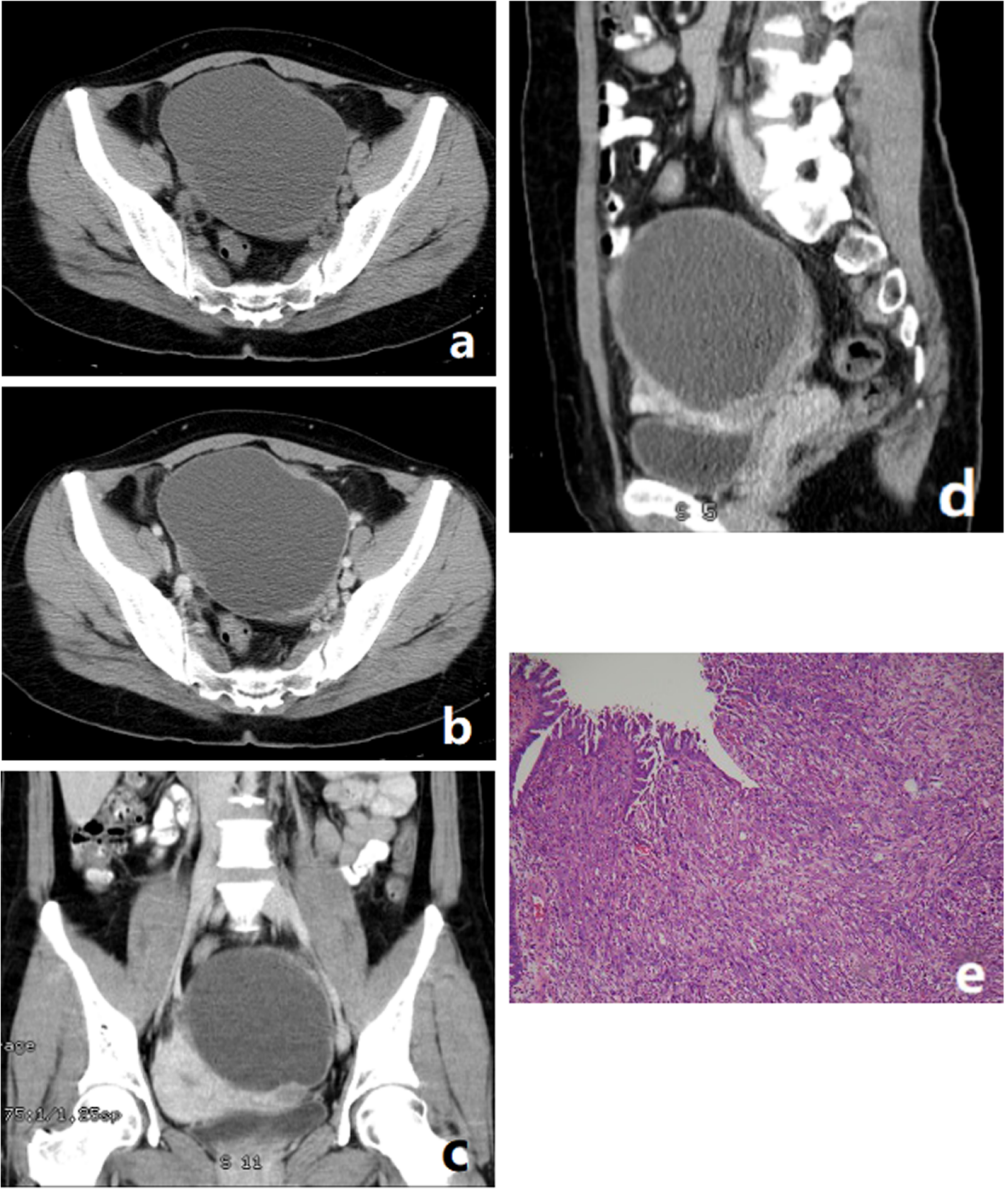
OCS in a 44-year-old woman with abdominal pain. **a** Axial CT showed a unilocular cystic mass with multiple mural nodules. **b**, **c**, **d** After the administration of contrast agent, the tumor parenchyma appeared moderate enhancement. **e** Photomicrograph showing a biphasic tumor with both carcinomatous and sarcomatous elements (HE20&10). The carcinomatous component was of glandular type. The sarcomatous part was composed of spindle-shaped cells with hyperchromatic pleomorphic nuclei. (HE 20&10)

All patients had unilateral tumors, four in the left ovary, one in the right ovary. All tumors were large bulky masses with the largest transverse diameter ranging from 11 cm to 14 cm. All the tumors were poorly defined, and no calcification was observed. The solid components of the tumors were iso-dense. MRI was performed in two cases. Hemorrhage was seen in the two tumors. The solid components of the two tumors demonstrated restricted diffusion (the average ADC value being 998 mm^2^/s and 1102 mm^2^/s, respectively). The tumor invaded the pelvic organs, peritoneal implants in case 5, pelvic peritoneal implants were observed in case 4 and case 1. In case 3, malignant cells were found in the right pleural and ascites. No extension to the pelvic organs, peritoneal implants and enlarged lymph nodes was observed in case 2. CT and MRI findings of five cases of OCS are summarized in Table 2, and representative cases are illustrated in Figs. 1, 2 and 3.

**Table 2:** The CT and MRI findings of four patients with ovarian carcinosarcoma

NO./side	Size (cm^3^)	Components	CT attenuation (solid)	SI (solid)			Intratumoral calcification/hemorrhage	Ascites/Pleural effusion	Enhancement (Solid)	Metastasis
				T1WI	T2WI	DWI/ADC, ×mm^2^/s				
1/R	11.5 × 8.1 × 12	Solid with-	Iso-	/	/	/	−/−	+/−	moderate	–
2/L	11 × 10 × 14	Multi-	Iso-	Slightly low	Slightly high	High/998	−/+	−/−	moderate	–
3/L	11.8 × 10.3 × 12.0	Solid with-	Iso-	/	/	/	−/−	+/+	moderate	+
4/L	11.1 × 9.3 × 9.0	Unilocular Cyst-	Iso-	/	/	/	−/−	−/−	moderate	+
5/L	9.0 × 9.3 × 8.0	Multilocular Cyst-	Iso-	Slightly low	Slightly high	High/1102	−/+	−/−	obvious	+

*SI* Signal intensity, *Multi-* Multilocular cyst mass with solid protrusion, *Solid with-* Solid with many cystic or necrosis areas, *Unilocular cyst-* Unilocular cyst with mural nodules, *Multilocular cyst-* Multilocular cyst with mural nodules, *ADC* The apparent diffusion coefficient; the symbol “+” denotes positive and “-” denotes negative

### Treatment and follow-up

Surgery was the primary treatment for all patients. According to the international federation of obstetrics and gynecology (FIGO) staging system, the five patients were classified as IIB, IC2, IV, IIB and IIIC, respectively. All five patients underwent total hysterectomy with bilateral salping-oophorectomy, peritoneal biopsies, omentectomy, and pelvic and paraaortic lymph node dissection. One patient died from recurrence and metastasis after 6 months. Three patients received 6 cycles of postoperative chemotherapy, had an uneventful postoperative period and are still alive, healthy and in follow-up, the longest follow-up time being 59 months. One patient is currently undergoing post-operative chemotherapy 1 month after operation and clinical stable.

### Histopathological findings

The immunohistochemistry results and pathology findings are summarized in Table 3. Microscopic examination of the five tumors showed a biphasic tumor with both carcinomatous and sarcomatous elements. There were some differences in malignant epithelial and sarcomatous elements between the five tumors.

**Table 3:** Histopathological features of ovarian carcinosarcoma

No.	Pathological diagnosis	Epithelial elements	Sarcomatous elements	Immunohistochemistry
1	Carcinosarcoma	Serous carcinoma	Chondrosarcoma	/
2	Carcinosarcoma	Serous carcinoma; Clear cell adenocarcinoma; Endometrioid carcinoma	Non-specific	CK (+)、CD10 (−/+)、P53 (−)、Ki-67 (−)
3	Carcinosarcoma	Serous carcinoma	Rhabdomyosarcoma	CK (+)、Vimentin (+)、S-100 (+)、MyoD1 (+)、Desmin (+)、P63 (+)、ER (−)、PR (−)、CA125 (+)、P53 (+)、Ki-67 (+++)
4	Carcinosarcoma	Mucinous carcinoma	Non-specific	CK (−)、Vimentin (+)、ER (−)、PR (+)、PAX8 (+)、WT1 (−)、Ki-67 (++++)、P53 (+)、CK7 (−)、CK20 (−)
5	Carcinosarcoma	Serous carcinoma	Rhabdomyosarcoma	P16 (+++)、PAX2 (−/+)、ER (+)、PR (−)、Ki-67 (80%+)、P53 (+++)、CK (+/−)、CK7 (+)、Vimentin (+)、E-cadherin (+/−); MyoD1(+)、Myoglobin (−); S-100 (+/−)、Desmin (+)。

## Discussion

Carcinosarcomas, also called malignant mixed müllerian tumors, occur commonly in the uterus. The occurrence in the ovary is an extremely rare event, and account for less than 1% of all ovarian cancers. Some studies believe that OCS occur most commonly in postmenopausal women of low parity and often present with disseminated disease at diagnosis with the average age was 65.5 years (range 55–77 years) [1, 8]. However, one patient in 40 years of age were represented by Dasgupta [7]. Menon et al. [9] showed the age at time of diagnosis of OCS varied from 33 to 70 years with a median age of 51 years. In our study, the average age of these patients was 45.4 years (range: 31–59 years).

Clinical features associated with OCS are nonspecific and related to tumor location, size, invasiveness. The symptoms include pelvic and / or abdominal pain, early satiety, bloating, abdominal distention, and gastrointestinal complaints [10]. Most patients present with symptoms of advanced stage disease, FIGO Stage III and IV [2, 10, 11]. In our study, clinical manifestations of two patients with advanced stage disease, FIGO Stage III C and IV were abdominal pain, weakness and weight loss. Of the remaining three patients with early stage disease, two were also abdominal pain and one was asymptomatic. This study supports the clinical manifestations of OCS are nonspecific.

CA 125 is a non-specific tumor marker usually associated with ovarian malignant tumors. In a study by Menon et al. [9] reported that CA 125 levels were raised in 9 out of the 12 cases of ovarian carcinosarcoma. Dai et al. [12]. reported a mean CA 125 level of 696.54 ± 314.06 U/mL in patients with OCS. Some studies showed over 90% of patients with OCS had elevated CA 125 level, with a preoperative level of > 75 U/mL associated with poor outcome [10, 11]. Different from these literature reports, it is interesting to note that in our study the positivity of CA 125 at diagnosis was present in four cases, and the mean value of CA 125 levels was 810.75 U/mL (range: 94.4–2158 U/mL), which was not correlated with poor prognosis. Similarly, one patient with the negative of CA 125 showed poor prognosis. According to our study, CA 125 is useful for screening or diagnosis of ovarian carcinosarcoma, maybe its elevation has no correlation with prognosis.

The increased serum levels of CA 153 have been established as a biomarker for breast cancer diagnosis since 1980s. Recently, a study of 19,789 clinical lab test results of serum CA153 levels found that patients suffering ovarian cancers had significantly increased median serum CA153 levels compared to that of healthy controls [13]. In our study, 80% (3/5) of patients had elevated CA153 levels. This study suggests that patients with OCS can show elevated CA 153 levels. A large study would be required to clarify the correlation between OCS and serum CA153 levels.

Many ovarian tumors manifest as large pelvic masses. Clinical, some studies showed that patients with OCS are more likely to present with a unilateral, large pelvic mass [4, 6, 13]. The radiological findings of OCS are not well known, as few studies have described the imaging feature of the disease. Daimon et al. [6] reported a case of this tumor showing a multilocular mixed cystic-solid masses with a diameter of 27 cm, and the solid part of the tumor was hemorrhage. Uçar et al. [4] reported a large solid, well-circumscribed mass of OCS measuring 18 cm in the largest transverse diameter. Pankaj et al. [14] showed a case of OCS with multiloculated mixed cystic-solid mass and the size was 10 × 6.8 cm. One case reported by Vernadakis et al. [3] showed a giant mass of 33 × 22 × 10 cm in size containing both solid and cystic elements. In our study, the five tumors were unilateral, cystic-solid masses, and the largest transverse diameter ranged from 11 cm to 14 cm, which was similar to reports indicating that the size of masses generally [3, 4, 6, 13, 14].

In the literature, OCS are highly aggressive tumors with rapid progression and poor prognosis [6, 15, 16]. Advanced stage at presentation, suboptimal surgical resection and older age are associated with poor prognosis [2]. Patients of OCS usually have advanced disease at the time of diagnosis, and about 75% of the cases present with widespread metastatic disease [stages III-IV] at the initial surgery [1, 10, 15, 16]. Due to the small number in our study, the five patients at the time of diagnosis were IIB, IIB, IC2, IV and III, respectively, and 80% (4/5) patients were younger than 52 years. The preferred treatment for most cases of OCS consists of cytoreductive surgery followed by platinum-based chemotherapy. Following primary surgical debulking, the consensus has been to recommend stage I to IV should have platinum-based chemotherapy in the treatment of OCS [10]. In our study, all the patients underwent maximal tumor reduction surgery, and three patients who survived and followed up were also treated with combination platinum-based chemotherapy. One patient is currently undergoing post-operative chemotherapy 1 month after operation and clinical stable.

In contrast to those published by Rauh-Hain, Morrow, and Harris et al. [17–20], our study found the data on long-term survival are more than the median defined by previous studies. In fact, only one patient was stage IIB and died 6 months after the operation. Three patients were stage IIB, IC2, and IV, respectively, and all are still alive, healthy and in follow-up (the mean months: 40 months, range from 9 to 59 months).

Microscopically, the influence of proportion of the malignant epithelial or sarcomatous component, as also heterologous vs homologous, on the disease of OCS progression is a matter of debate. Some early studies have reported that the presence of heterologous sarcomatous elements is associated with a poor prognosis [10, 11, 21]. Some studies in recent years have noted that the sarcomatous element (heterologous vs homologous) has no clear influence on patient outcome [1, 10, 22–25]. In addition, Athavale et al. [25] stated that tumors with stromal predominance and serous epithelial component have worse prognosis. In our study, three patients with the heterologous sarcomatous and / or serous epithelial component in the tumor had a good prognosis.

Like in every study, our study also has some limitations. First, patient selection bias existed because of the retrospective nature. Second, our sample size was small. A larger study would be required to definitively establish the characteristic features of OCS. Third, long term follow-up is highly advised.

## Conclusions

OCS are rare tumors, the clinical and imaging features lack specificity. Our preliminary study demonstrates that the levels of CA125 and CA153 of patients with OCS are elevated, and the tumors presented as unilateral large cystic-solid masses with moderately or highly enhanced, a relatively higher ADC value in the solid component. Although the diagnostic performance of any feature alone is not sufficient for diagnosis, the detailed clinical and imaging features may be helpful to improve the familiarity of this rare tumor. Given its aggressive nature and poor prognosis, OCS require treatment by radical surgical resection and careful follow-up with CT or MR.

## Materials and methods

### Patients

This retrospective case series included five patients who were diagnosed with ovarian carcinosarcoma from June 2013 to August 2019. Patients were identified from the case records of the hospital.

This retrospective study was approved by Service Ethics Committee of Women’s Hospital, Zhejiang University School of Medicine (Zhejiang, China). All patients provided written informed consent to participate in this study.

### Image acquisition

All five patients underwent CT scans, two patients underwent MRI scans.

CT scans were obtained with 16-detector row scanner (GE Medical Systems, Milwaukee, WI). The main imaging parameters were as follows: 5 mm section thickness reconstructions, 25 cm field of view, 120 kA tube voltage, 300 mA current, and a 512 × 512 matrix. The contrast medium injected was iopromide (Ultravist, 300 mg/mL) with a dose of 1.5 mL/kg and an injection rate of 2.5 mL/s who underwent contrast-enhanced CT. None of the patients were allergic to the iodine contrast medium. Contrast-enhanced CT scans were started 50 to 60 s after the administration of the contrast agent.

MR examinations were performed using on a 1.5-T scanner (Signa HDxt; GE Healthcare, Milwaukee, WI) with a phased-array abdominal coil. The patients laid in a supine position and breathed freely during acquisition. The sequences were obtained as follows: axial spin echo (SE) T1-weighted imaging (T1WI) [time of repetition (TR) / time of echo (TE), 340 ms/10 ms]; axial fast SE T2-weighted imaging (T2WI) with and without fat saturation (TR/TE, 8000 ms / 83 ms and 4000 ms / 98 ms, respectively); and sagittal and coronal fast SE T2WI (TR/TE, 8000 ms / 83 ms). Diffusion-weighted imaging (DWI) was obtained in axial planes at b values of 0, 800 s/mm^2^ (TR/TE 4600 ms / 72 ms). The triple-phase dynamic MR-enhanced scans were performed in the axial, sagittal, and coronal planes immediately after the intravenous administration of Gadopentetate dimeglumine (Magnevist; Bayer Schering, Guangzhou, China) at a dose of 0.2 mmol/kg of body weight and a rate of 2 to 3 mL/s. The scanning parameters were as follows: 5 mm slice thickness, 1.2 mm gap, 256 to 320 × 256 to 320 matrix, 250 to 296 mm × 250 to 340 mm field of view and four excitations. The scanning range was from the inferior pubic symphysis to the renal hilum and extended beyond the dome of the tumor in cases with huge masses.

### Image analysis

The imaging characteristic of the tumors were retrospectively evaluated by two trained radiologists in consensus. The imaging findings were evaluated as follows: 1) laterality, shape and size; 2) cystic or solid components; 3) density (hypo-, iso-, and hyper-density, referring to the density of the myometrium); 4) signal intensity (SI) (the signal similar to inner outer myometrium and fat was considered low, moderate and high, respectively); 4) enhancement (mild, moderate or obvious by referencing those of the junctional zone and outer myometrium); 5) amount of ascites and pleural effusion (none, mild, moderate, and severe); 6) apparent diffusion coefficient (ADC) value as measured on ADC maps, a circular region of interest (ROI) of at least 1 cm^2^ was placed at targeted areas with the possibly lowest ADC values in the solid components of the tumor, by referring to conventional MR imaging and avoiding areas such as hemorrhage, necrosis and major vascular structures. At least three measurements were obtained and averaged.

### Pathological analysis

In all cases, interpretation of frozen sectioning performed during surgery suggested OCS. The final diagnosis was deferred to the analysis of permanent section. The histological technique consisted of routine hematoxylin and eosin (H&E) staining and immunohistochemical evaluation.

## Data Availability

All data were included in this article.

## Abbreviations

OCS: Ovarian carcinosarcoma
CT: Computed tomography
MRI: Magnetic resonance imaging
MMMT: Malignant mixed müllerian tumor
DWI: Diffusion-weighted imaging
ADC: Apparent diffusion coefficient
SI: Signal intensity
ROI: Circular region of interest
CA125: Cancer antigen 125
CA153: Cancer antigen 153
CA199: Cancer antigen 199
FD: Follow-up
FIGO: The international federation of obstetrics and gynecology staging system
